# An Attention-Enhanced Multimodal Hybrid Model for Skin Cancer Diagnosis Using Imaging and Clinical Data

**DOI:** 10.3390/biomedicines14071532

**Published:** 2026-07-08

**Authors:** Fatima Erik Dogan, Merve Kesim Onal, Harun Bingol, Sercan Yalcin, Muhammed Yildirim

**Affiliations:** 1 Department of Dermatology, Elazig Fethi Sekin City Hospital, Elazig 23300, Türkiye; fatimaerik@windowslive.com; 2 Department of Computer Engineering, Malatya Turgut Ozal University, Malatya 44210, Türkiye; merve.kesim@ozal.edu.tr; 3 Department of Software Engineering, Malatya Turgut Ozal University, Malatya 44210, Türkiye; 4 Department of Computer Engineering, Adiyaman University, Adiyaman 02000, Türkiye; svancin@adiyaman.edu.tr; 5 Department of Artificial Intelligence and Data Engineering, Firat University, Elazig 23119, Türkiye; muhammedyildirim@firat.edu.tr

**Keywords:** channel attention, deep learning, FT-transformer, skin cancer, ViT

## Abstract

**Background/Objectives**: Skin cancer is one of the most common diseases worldwide, with a high mortality rate. Due to its ability to metastasize, the disease can progress to more serious stages over time. This article proposes a hybrid model based on feature engineering that will play a critical role in the early diagnosis of the disease. **Methods**: The developed model in this paper utilizes the well-known Vision Transformer (ViT) and Convolutional Neural Network (CNN) models for feature extraction from images in the dataset, while the FT-Transformer, Excel Former, SAINT, GRANDE, PTaRL, and TabTransformer architectures are used for feature extraction from clinical data. Furthermore, this study was developed using a very large pool of classifiers, including 13 classifiers. Fine-tuning was applied to improve the performance of the developed model. Channel attention mechanisms were incorporated into the study to ensure that the proposed model focuses on the diseased area. The PAD-UFES-20 dataset was used during the experiments. Class weighting was applied to the proposed model to prevent class-based imbalance in the PAD-UFES-20 dataset. **Results**: Six distinct CNN and four distinct ViT models were compared to the developed model. The developed model achieved a highly competitive Area Under the Curve (AUC) rate of 96.41%. The study was conducted using a dataset containing both clinical and imaging data. **Conclusions**: The proposed model is thought to help dermatologists diagnose skin cancer.

## 1. Introduction

Skin cancer, which is divided into two types, melanoma and non-melanoma, is known to be the most common type of cancer worldwide. It is known that individuals with fair skin have a higher risk of developing this disease than those with darker skin. Individuals with red hair and freckling are also known to have a higher risk of developing skin cancer compared to other individuals. Natural preventative measures include avoiding direct sun exposure and refraining from artificial skin darkening treatments such as tanning beds [1,2,3]. Skin cancer is known to be caused by a variety of variables, including genetics, changes in recreational activities, and social lifestyle choices, in addition to direct sun exposure. Using ultraviolet (UV) protective creams is one of the most important daily measures to take to protect against skin cancer. Additionally, protecting yourself from the sun’s rays between 11:00 AM and 4:00 PM, when the sun’s rays are at their strongest, is another beneficial measure [4]. Melanoma is known as the most dangerous type of skin cancer. Early diagnosis of this type of cancer is known to help improve the prognosis. Otherwise, in advanced stages, the disease can metastasize to other organs, which can result in the patient’s death [5].

A specialist will normally detect skin cancer by analyzing tissue removed from the affected area in a lab; this usually manifests as a nevus. The laboratory tissue examination process is prone to error, costly, and time-consuming. In particular, the latest technological advancements in image, sound, text, and signal processing have enabled the effective use of computer-aided systems in many fields such as aviation [6], transportation [7], healthcare [8], chemistry [9], and energy [10]. The main reason for the use of computer-aided systems as a decision support system in the medical field in recent years is known to be their high accuracy rate [11]. A decision support system specifically developed for skin cancer, specializing in early diagnosis, could be beneficial in detecting the disease before it progresses further [12,13]. There are many types of skin cancer. Differentiating between these types requires considerable expertise. The knowledge and experience gained by specialists in diagnosing the patient directly affect the treatment process, and it is known that a possible misdiagnosis can have serious effects on the course of the disease. Because learning systems operate with labeled data based on expert knowledge, they have been widely used in the medical field in recent years due to their high diagnostic performance. Manual methods are time-consuming, prone to errors, and expensive. Today, systems that assist the expert with high accuracy, fewer errors, and lower cost are in high demand. In this context, a high-performance multimodal hybrid model that will assist the expert in diagnosing skin cancer with less time and cost is proposed within the scope of this study.

Deep learning techniques have emerged in recent years as concepts intertwined with feature engineering. The understanding of searching for next-generation solutions to existing problems by processing and examining deep features extracted from images, sound, text, or signals is quite popular today. Unlike traditional methods, this approach aims to search for and develop methods based on the deep features of the data in the dataset. The PAD-UFES-20 dataset, which is accessible to the public, was used in this investigation. The fact that the dataset used in this study contains both clinical and imaging datasets makes it different from previous studies in the literature. In order to create a decision support system for the early diagnosis and detection of skin cancer, this study selected useful elements from both clinical and imaging data. In order to improve the disease diagnosis performance of the proposed deep hybrid model in this study, fine-tuning was applied to the training data. Deep features extracted from ViT and CNN architectures were combined. Channel attention was applied to the resulting feature map. After this stage, a feature map was created by combining the features obtained from clinical data with the features obtained from image data. Channel attention was reapplied to improve diagnostic performance in the growing feature map by incorporating clinical data. The use of two attention mechanisms in the model was implemented to ensure that the model focuses on disease-related features and to improve diagnostic performance. The feature map, which underwent a double attention process, was subjected to a class weighting operation to balance the class-based weights in the dataset. This step aimed to prevent problems that might arise from class-based imbalances. Thirteen distinct machine learning classifiers were used to classify the final feature map in the suggested model.

### 1.1. Contribution and Novelty

This paper proposes a hybrid model for diagnosing skin cancer and related diseases by processing both image and clinical data. The combined use of disease-related images and clinical data aligns with real-world disease scenarios. This study establishes a reproducible pre-processing pipeline. The innovative aspects of this study and its contributions to the literature are briefly listed below.

- A hybrid model based on CNN, ViT, and channel attention mechanisms has been developed, and feature extraction has been performed automatically using CNN and ViT architectures. These features were then classified into 13 different classifiers.
- ConvNeXt-V2-Base and ViT B-16 transformer designs were used to extract features from image data in the first stage of the suggested model. Then, feature extraction was performed on clinical data using the FT Transform architecture. The obtained features were combined to create a feature map. After processing this feature map through the channel attention module, class weighting was applied. Finally, the feature map was classified by classifiers.
- The model was fine-tuned to increase the diagnosis precision of skin cancer and associated conditions.
- The AUC value of the hybrid model proposed in this study was obtained as 96.41%.

### 1.2. Related Works

When examining the methods using the PAD-UFES-20 dataset, it was observed that almost all studies attempted to classify the images included in the dataset without processing the clinical data. In this study, we propose a system that processes both the images and clinical data in the dataset. Therefore, studies that only classify images are included in this section. In this study, many studies in the literature on the early diagnosis and detection of skin cancer were examined. It was generally observed that the classification of images related to the disease was carried out using various methods.

Craythorne and Al-Niami stated that skin cancer is the most common type of cancer in the world and its incidence is increasing every day. Although UV exposure is a significant cause of the disease in general, they state that genetic predisposition, in other words, a low Fitzpatrick phototype, is also an important factor [14].

Javed et al. extracted features from images using the ConvMixer architecture, which is a CNN architecture that works like transformers but keeps the disease image size and resolution constant across all layers, and performed classification in CatBoost, XGBoost, and LGBM classifiers. It is stated that synthetic data was generated using the SMOTE technique. It is emphasized that the class imbalance in the original dataset was overcome by data multiplexing. It is stated that the highest performance was obtained with the CatBoost classifier, with the macro-AUC metric at 94% and the macro-F1 metric at 88% [15].

Azeem et al. propose a 4-layer CNN architecture called Skin-LesNet for classifying skin cancer. They enhanced the images in the PAD-UFES-20 dataset to overcome class imbalance. They conducted experiments with the 3-class modified PAD-UFES-20 dataset and reported achieving an accuracy of 96% [16].

Bhargavi and Balakrishna, in their study, extracted features from some CNN architectures to classify skin cancer and obtained a feature map by combining these features. Then, they tested this architecture on the PAD-UFES-20 dataset, where they performed data augmentation. The researchers reported an accuracy rate of 96.17% in the study [17].

Oztel et al., in their study, implemented a classification application for skin cancer and monkeypox disease using the ResNet18 architecture running on a mobile device. The authors stated that they achieved 74.62% accuracy and 65.90% F1-score in the 6-class PAD-UFES-20 dataset. They stated that they used data enhancement techniques to eliminate class imbalance in the dataset [18].

Wang et al. used the Dense-Net121 architecture for skin cancer classification in their study. By adding 1063 disease images that they extracted themselves to the 6-class PAD-UFES-20 dataset, the authors increased the number of images in the dataset to 3361. They stated that they obtained an accuracy of 91.86% and an AUC of 98% [19].

Khan et al. stated that they used the CNN-based EfficientNetB3 architecture together with the Extreme Gradient Boosting (XGBoost) architecture to classify skin cancer. The authors applied data enhancement techniques to improve the performance of the model they proposed with the community learning approach on the PAD-UFES-20 dataset. It is stated that evaluating clinical data together with skin lesions has a positive contribution to the accuracy of diagnosis. It is stated that the proposed model obtained 78% accuracy and 83% AUC in diagnosing the disease [20].

Sathvika et al. developed an application for the diagnosis and classification of skin diseases using a support vector machine and CNN-based AlexNet architecture with dual pipelines. During the experiments, they performed data multiplexing using a 5-class PAD-UFES-20 dataset. It is stated that the number of images per class was increased to 5 times the original number. Preprocessing and segmentation operations were performed on the skin lesion images. After the bisectional feature extraction stage, classification was performed using the SVM model. It is stated that the proposed method achieved 98.10% accuracy [21].

Bouzon et al. stated that they performed sentence embedding operations on the MetaBlock architecture. It is stated that by integrating metadata representation into the model, the robustness of the model in diagnosing the disease against missing data has been increased. In addition, it is stated that an accuracy value of 70.2% was obtained in the PAD-UFES-20 dataset [22].

### 1.3. Organization of Paper

The paper continues with the Materials and Methods section, which examines the dataset, models, and methods used in the paper. Section 3 presents the results obtained in the paper. Section 4 contains the discussion section, and the paper concludes with the conclusion section.

## 2. Materials and Methods

### 2.1. Dataset

In the study conducted, the PAD-UFES-20 dataset was used for the detection of skin cancer [23]. The PAD-UFES-20 dataset consists of 1641 skin lesions and 2298 images collected from 1373 patients. This dataset contains a total of 6 classes, including 3 skin diseases and 3 skin cancers. These classes are Actinic keratosis (ACK), Basal cell carcinoma (BCC), Seborrheic keratosis (SEK), Squamous cell carcinoma (SCC), Malignant melanoma (MEL), and Melanocytic nevus (NEV). In this dataset, a total of 26 attributes, 21 of which are clinical features, such as the patient’s age, gender, smoking status, and presence of pain or bleeding, are included as clinical data in each image. The class-wise distribution of the dataset is given in Table 1.

**Figure 1:**
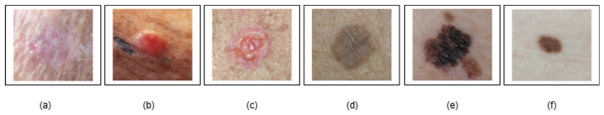
Sample images from the PAD-UFES-20 dataset (**a**) ACK (**b**) BCC (**c**) SCC (**d**) SEK (**e**) MEL (**f**) NEV.

The resolution, size, and lighting properties of the photographs in the dataset vary since they were taken using various equipment [23]. Figure 1 displays sample photos from the dataset.

### 2.2. Overall Proposed Model

This study proposes a multimodal deep learning architecture for the classification of skin lesions. The proposed approach includes feature extraction based on image and clinical metadata, weighting of features with attention mechanisms, and classification. In all models used in the study, class weighting was applied to reduce the effect of the inter-class imbalance problem in the PAD-UFES-20 dataset. This method regulates the loss function by assigning higher weights to samples belonging to minority classes. Thus, the model is prevented from overfitting to the majority class, and the aim is to increase the classification success in minority classes [24]. The proposed class imbalance problem in this investigation was resolved using a class-balanced loss function depending on the number of effective samples, as suggested by Yin Cui et al. [25]. The weight *W_i_* for every class is determined using this approach as follows:

(1) $$W_{i}=\frac{1-\beta }{1-\beta^{n_{i}}}$$

In Equation (1), $W_{i}$ represents the weight value calculated for each class, $n_{i}$ represents the number of samples in the i-th class, and β is the hyperparameter that controls the increase in the effective sample size. Furthermore, the calculated $W_{i}$ values are limited to the range [0.25, 2] to prevent excessive suppression of dominant classes.

For the image modality, two complementary deep learning architectures were used in parallel. In model selection, two main architecture groups were evaluated: CNN architectures that learn local features hierarchically across layers (ConvNeXt V2 Base [26], DenseNet 121 [27], EfficientNet B4 [28], Inception V3 [29], MobileNetV3 [30], Resnet50 [31]) and Transformer architectures that can model global information by capturing relationships between locations (ViT B-16 [32], ViT B-32 [33], DeiT B-16 [34], Swin Base [35]) [36,37,38]. From both groups, ConvNeXt-V2-Base from the CNN group and ViT B-16 from the Transformer group, which exhibited the highest performance on the PAD-UFES-20 dataset, were selected. Both models were initialized with pre-trained weights on ImageNet and subjected to fine-tuning to learn features specific to the PAD-UFES-20 dataset. Fine-tuning is used to retrain a deep learning model trained on large datasets with new data specific to the target task. In this approach, the model is trained by replacing the last layers of the model with layers suitable for the new task. Thus, while benefiting from the general feature extraction skills the model gained in previous training, target-specific features are also learned [39].

After fine-tuning, feature vectors of 1024 and 768 dimensions were obtained from the average pooling layer of the ConvNeXt-V2-Base and ViT B-16 models, respectively; these vectors were combined to obtain a 1792-dimensional feature vector. Then, this vector was transferred to the SE-Net attention mechanism, which weights informative and distinctive features [40]. In the 1792-dimensional weighted feature vector, which is the output of the SE block, dimensionality reduction was applied to reduce computational cost, lower the risk of over-learning, and maintain intermodal balance by reducing the dimensionality difference to a reasonable level with the 256-dimensional FT-Transformer output obtained from the clinical data section. For this stage, a block consisting of a Linear layer, Batch Normalization, and ReLU activation function was used, and the vector was reduced to 1024 dimensions. The Feature Tokenizer Transformer (FT-Transformer) architecture was used for the clinical metadata modality. With the model, structured patient information such as age, gender, smoking status, and lesion region in the PAD-UFES-20 dataset was transformed into a 256-dimensional feature vector. FT-Transformer is a deep learning architecture designed specifically for tabular data modeling tasks, capable of learning complex feature interactions and long-range dependencies. Unlike traditional Transformer models, it treats each feature as an independent token and learns relationships between features in an order-independent manner [41].

**Figure 2:**
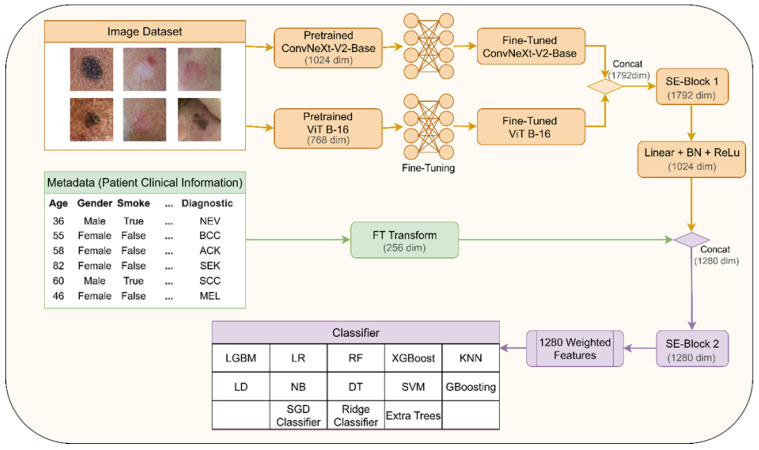
Overall chart of proposed model.

A 1024-dimensional feature vector obtained from the image modality and a 256-dimensional feature vector obtained from the clinical metadata modality were combined to create a 1280-dimensional feature vector. Then, the combined features from the two modalities were transferred to a second Squeeze-and-Excitation (SE) block in order to reweight their significance levels [42]. In the final stage, the weighted feature vector was given as input to 13 different machine learning classifiers, namely LightGBM (LGBM) [43], Logistic Regression (LR) [44], Random Forest (RF) [45], XGBoost [46], K-Nearest Neighbors (KNN) [47], Linear Discriminant Analysis (LD) [48], Naive Bayes (NB) [49], Decision Tree (DT) [50], Support Vector Machine (SVM) [51], Gradient Boosting (GBoosting) [52], SGD Classifier [53], Ridge Classifier [54] and Extra Trees [55], and compared. The general flow of the proposed architecture is given in Figure 2.

A series of preprocessing steps was used for all architectures employed in the study. Image data was rescaled to 256 × 256 pixels before being fed into the model as input, followed by a random crop to 224 × 224 pixels. To increase data diversity and prevent overfitting, the training set was subjected to random horizontal and vertical flipping, color jitter (including brightness, contrast, saturation, and hue adjustments), ±20° rotation, Gaussian blurring, and random erasing. The test set was subjected to only 224 × 224 resizing. All images were also normalized. Clinical metadata features were completed by filling in missing values with the median and scaled using StandardScaler. The model was divided using a stratified split method, with 80% training and 20% testing. The hyperparameters and experimental settings utilized during the fine-tuning phase are summarized in Table 2. The model weights yielding the highest accuracy on the validation set were saved and employed for the final architecture.

## 3. Results

The results of experiments conducted to evaluate the performance of the proposed model are presented in this section. The study also presents AUC and ROC curves. AUC represents the area under the ROC curve and measures the model’s ability to discriminate between classes. Thanks to this feature, it is considered a more reliable performance metric when compared with metrics such as accuracy in datasets where inter-class imbalance is significant [56]. The results were obtained using a computer consisting of an Intel(R) Core (TM) Ultra 9 275HX processor, 32 GB RAM, and an NVIDIA GeForce RTX 5070 Ti graphics card with 12 GB of RAM. On the software side, Python 3.12.3 and CUDA Toolkit 12.6 were used.

### 3.1. Results of Pre-Trained Models

To determine the models to be used as the basis for the proposed model, various CNN and ViT models for images in the PAD-UFES-20 dataset and tabular deep learning models for clinical data were compared. For the image modality, the commonly used architectures in the literature, ConvNeXt V2 Base, DenseNet 121, EfficientNet B4, Inception v3, MobileNetV3, Resnet50, ViT B-16, ViT B-32, DeiT B-16, and Swin Base, were trained. The performance results of the pre-trained CNN and Transformer models are given in Table 3.

When Table 3 is examined, it is seen that among CNN architectures, the ConvNeXt V2 Base model exhibits the highest performance with 0.7652 accuracy, 0.7622 macro F1 score, and 0.9462 AUC values. Among Transformer architectures, the ViT B-16 model stands out with 0.7696 accuracy, 0.7643 macro F1 score, and 0.9367 AUC values. In line with these results, ConvNeXt V2 Base from the CNN group and ViT B-16 models from the Transformer group were selected as image encoders for the hybrid architecture.

**Figure 3:**
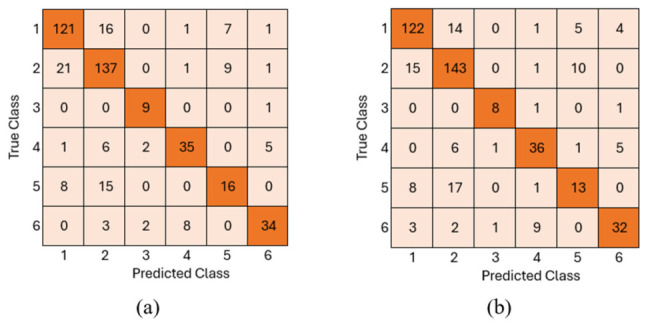
(**a**) Confusion matrix of the ConvNeXt V2 Base model. (**b**) Confusion matrix of the ViT B-16 model.

The confusion matrices of the highest-performing ConvNeXt V2 Base and ViT B-16 architectures are given in Figure 3. Classes numbered 1 to 6 in the confusion matrices represent ACK, BCC, MEL, NEV, SCC, and SEK skin lesion types, respectively.

Figure 3 shows that both models generally successfully distinguish between classes. The ConvNeXT V2 architecture’s accurate classification of 121, 137, and 34 examples in classes 1, 2, and 6, respectively, demonstrates a high recognition success rate in these classes. Similarly, the number of correct classifications is significantly higher in classes 3, 4, and 5 compared to other cells. Furthermore, some confusion is observed between classes. For example, 21 examples belonging to class 2 were predicted as class 1. Similarly, 15 examples belonging to class 5 were classified as class 2, and 8 as class 1, suggesting that these classes may share similar characteristics. The relatively low level of confusion in class 3 with other classes indicates that the model effectively learned the distinctive features specific to this class. The ViT B-16 architecture’s accurate classification of 122 and 143 examples in classes 1 and 2, respectively, demonstrates the model’s high recognition success in these classes. Similarly, the number of correct classifications in classes 3, 4, 5, and 6 is significantly higher compared to other cells. Furthermore, the low level of mixing in class 3 with other classes proves that the model has learned the distinctive features specific to this class.

**Figure 4:**
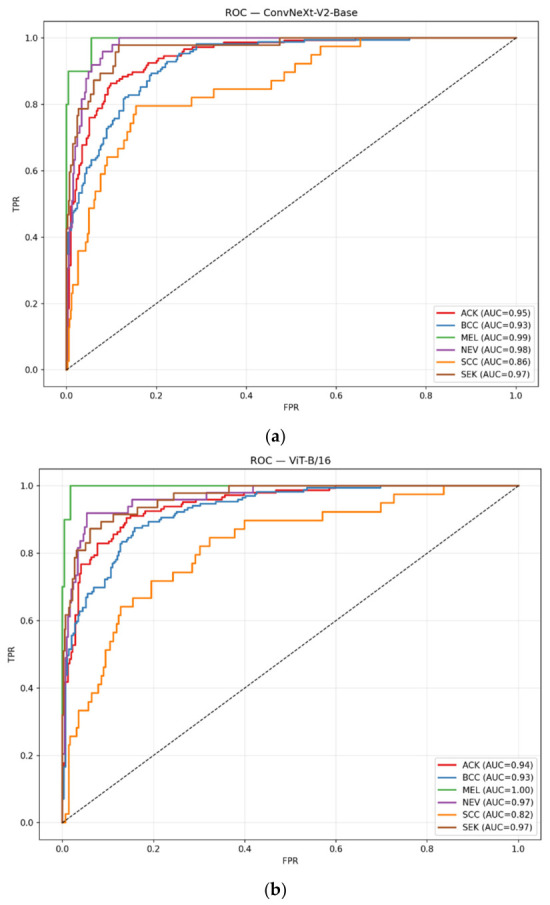
(**a**) ROC curve of the ConvNeXt V2 Base model, (**b**) ROC curve of the ViT B-16 model.

The ROC curves of the highest performing ConvNeXt V2 Base and ViT B-16 architectures are given in Figure 4.

When Figure 4 is examined, the curves clustered in the upper left indicate that the model is suitable for the problem and demonstrates high performance. Both the ConvNeXt V2 Base and ViT B-16 ROC curves show that the lowest AUC value is in the SCC class, while the highest AUC value is in the MEL class.

For the clinical metadata modality, the FT-Transformer, ExcelFormer, SAINT, GRANDE, PTaRL, and TabTransformer architectures, designed to work with tabular data, were trained using structured patient information from the PAD-UFES-20 dataset. The performance results of the trained models are given in Table 4.

**Figure 5:**
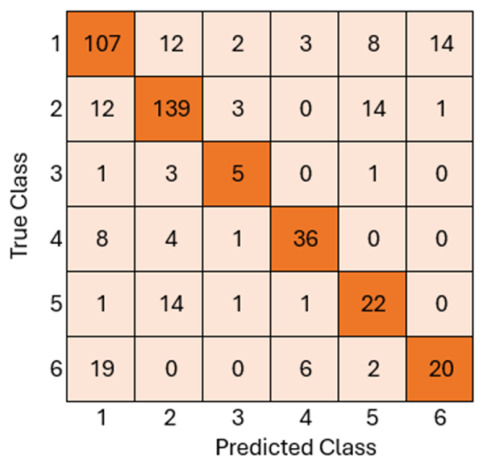
Confusion matrix of the FT-Transformer model.

With an accuracy of 0.7152, a macro F1 score of 0.7144, and an AUC of 0.9017, Table 3 demonstrates that the FT-Transformer model performed the best of all the models. ExcelFormer was the second-highest architecture with an accuracy of 0.713 and an AUC of 0.8919. The SAINT and GRANDE models showed similar performance, while the PTaRL and TabTransformer models lagged behind the other models in both accuracy and AUC. Based on these results, the FT-Transformer architecture was selected for the metadata modality. The confusion matrices of the highest-performing FT-Transformer and ExcelFormer architectures are given in Figure 5.

**Figure 6:**
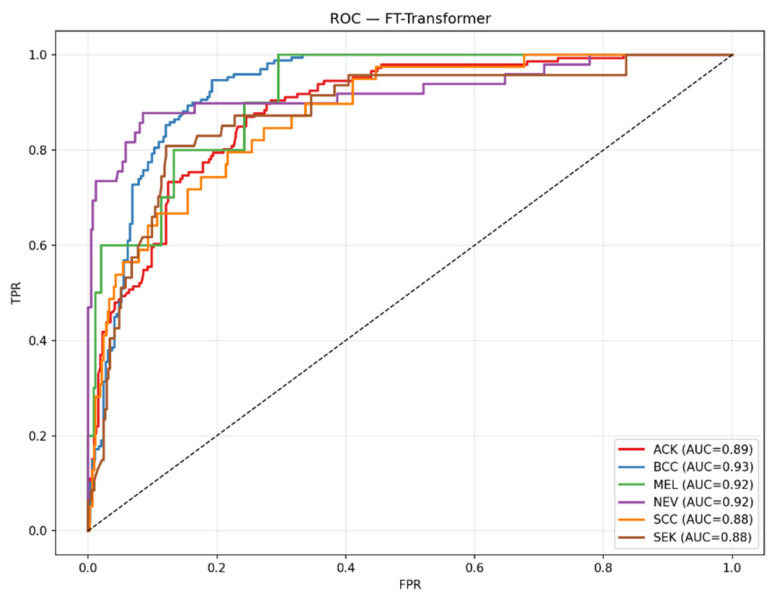
ROC curve of the FT-Transformer model.

Figure 5 shows that 139 metadata items in the BCC class were correctly predicted, while 12 in the ACK class, 3 in the MEL class, 14 in the SCC class, and 1 in the SEK class were incorrectly predicted. The ROC curve of the FT-Transformer architecture showing the highest performance is given in Figure 6.

When Figure 6 is examined, the performance in the SEC and SCC classes is similar but lower than that of other classes. Furthermore, the class-based performance of the FT-Transformer architecture indicates that all classes are generally distinguishable, as evidenced by the clustering of the ROC curves in the upper-left corner of the graph.

### 3.2. Results of Proposed Model

The 1280-dimensional weighted feature vector obtained from the proposed multimodal architecture was fed as input to thirteen different machine learning classifiers, and the models were evaluated comparatively. The aim of this comparison was to determine the classifier that best represents the feature vector and maximizes classification performance. The results are presented in Table 5.

**Figure 7:**
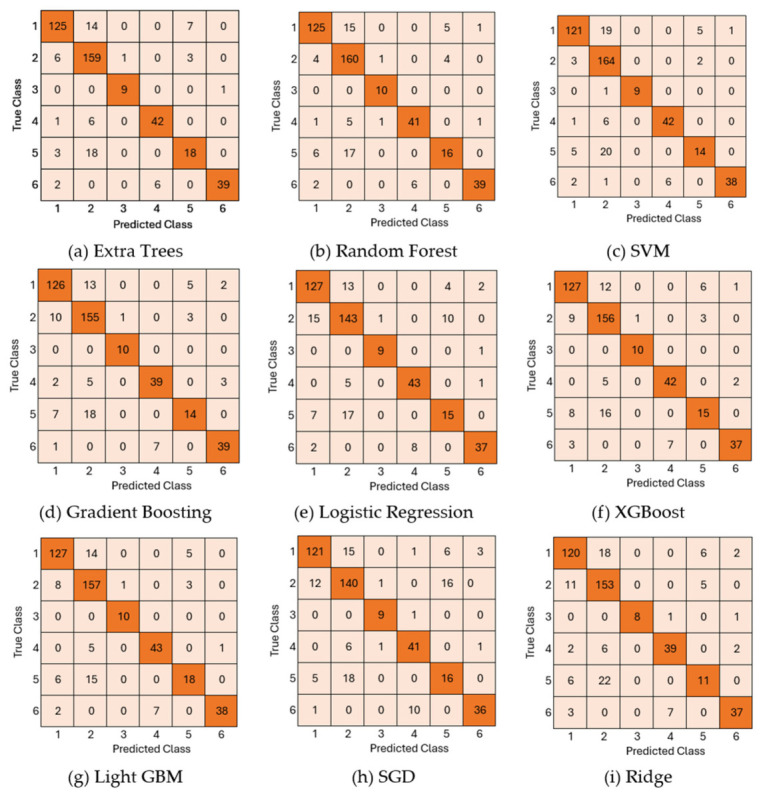
Confusion matrices of 9 models showing high AUC performance.

Table 5 shows that the Extra Trees model exhibits the highest AUC performance with an accuracy of 0.8522, a macro F1 score of 0.8486, and an AUC value of 0.9641. The Random Forest model ranks second in terms of AUC with an accuracy of 0.85, a macro F1 score of 0.8443, and an AUC value of 0.9603. Light GBM achieved the highest accuracy value (0.8543) while exhibiting a competitive performance in terms of AUC with 0.9476. The Linear Discriminant, Naive Bayes, and Decision Tree models showed lower accuracy and AUC performance compared to other classifiers. When all metrics are evaluated together, the Extra Trees model is seen to be the most balanced and high-performing classifier. The confusion matrices of the 9 models showing high AUC performance are given in Figure 7.

Figure 7 shows that 13 different classifiers exhibit balanced performance in both classes with a low and a high number of images. The class weighting application minimizes the risk of misclassification caused by class imbalance. In particular, the Extra Tree classifier, which showed the highest performance, correctly predicted 159 out of 169 test images in the BCC class, which has the highest data volume. This demonstrates Extra Tree’s strong discrimination capability on the dataset. Even in the MEL class, which has the least representation, it showed a very strong discriminatory ability. It correctly predicted 9 out of 10 test images, incorrectly predicting 1 as the SEK class.

**Figure 8:**
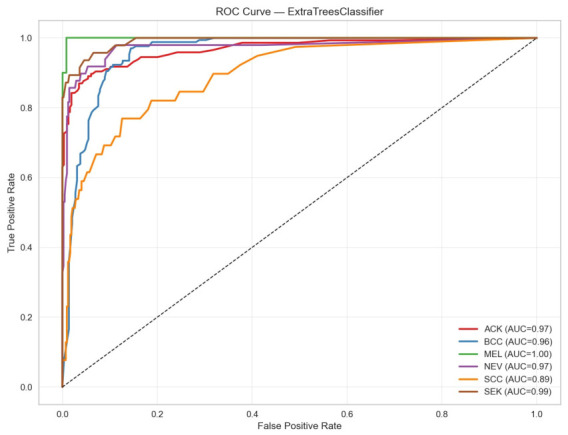
ROC curve of the proposed model.

Examining the ROC curve given in Figure 8, it is understood that the proposed model shows high success in all classes of skin lesions in the Extra Trees Classifier. The clustering of the curves in the upper left corner indicates that the model can successfully distinguish between different classes. Among the 6 different classes, the highest success is observed in the MEL class with AUC = 1.00. From a clinical perspective, since early and accurate detection of melanoma is vital, this result reveals a significant advantage of the model.

To compare the contributions of the SE block, results were also obtained using the Convolutional Block Attention Module (CBAM) block. The results obtained with the addition of the CBAM block are presented in Table 6.

**Figure 9:**
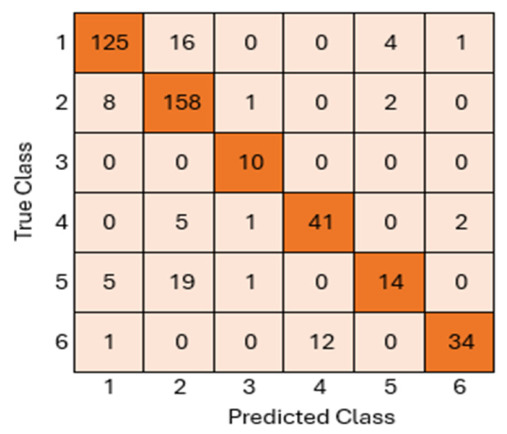
Logistic Regression confusion matrix after CBAM integration.

**Figure 10:**
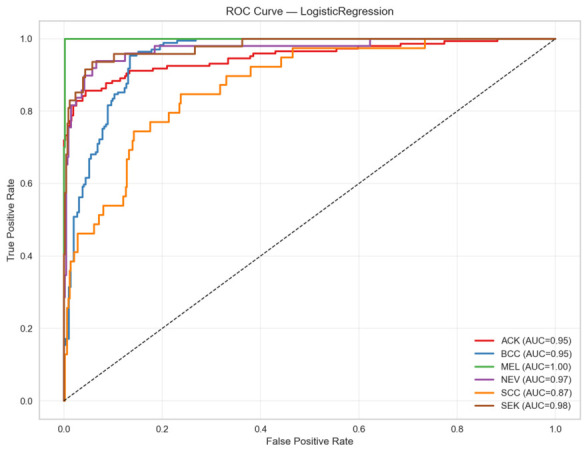
Logistic Regression ROC curve after CBAM integration.

AUC values obtained after CBAM integration showed a slight increase in 8 out of 13 classifiers and a slight decrease in 5 when compared to the original SE-based architecture. The AUC value of the Extra Trees classifier, selected as the most successful model with the SE-based architecture, decreased from 0.9641 to 0.9617 with CBAM regression. This finding indicates that the addition of CBAM did not affect the overall discriminative power. However, accuracy results obtained after CBAM integration show a performance decrease in 8 out of 13 classifiers when compared to the original SE-based architecture. In the best-performing models (Extra Trees, Random Forest, Light GBM, XGBoost), CBAM caused a 1–3 point decrease compared to SE. The complexity matrix of the Logistic Regression model, which achieved the highest performance after CBAM integration, is given in Figure 9, and the ROC curve is given in Figure 10.

## 4. Discussion

Skin cancer has shown an increasing trend worldwide in recent years. MEL is known to be particularly dangerous. Early diagnosis is frequently discussed in the literature as having a positive impact on treatment options, preventing the spread of the disease to other organs. To prevent diagnostic variability depending on the specialist examining the image, this study proposes a hybrid model aimed at mitigating human error. Literature review reveals that dermoscopic images are generally used to diagnose this disease. This study considers both clinical data and images of the disease. This technique, highly relevant to real-world scenarios, demonstrates that clinical data is as important as images in defining the disease.

The competitive performance of the proposed model in comparisons with similar studies and existing pre-trained models highlights the potential for integrating the approach into clinical decision support systems. The results support the idea that deep learning methods based on image and clinical data can be used as a preliminary decision support framework in the early detection of skin cancer. A detailed comparison with relevant studies is given in Table 5.

Table 7 demonstrates that, compared to similar studies in the literature, the suggested methodology yields more successful outcomes. This suggests that more distinctive representations in multi-class skin cancer classification problems can be obtained by combining The power of CNNs to extract local features was used in conjunction with Vision Transformer and attention mechanisms. Additionally, a weighted class approach was employed to address dataset imbalance. Results on a six-class, large-scale dataset demonstrate that while some studies with fewer classes have reported higher accuracy values, the proposed model provides a more suitable and generalizable structure for complex and multi-class datasets. When other classes were examined, the proposed model achieved an AUC of 0.97 in the ACK and NEV classes and 0.96 in the BCC class. These values indicate that the model is quite successful in differentiating between benign and malignant lesions and has a strong generalization ability. The fact that the ROC curves are quite far from the diagonal reference line confirms that the proposed model’s performance is far superior to random classification. However, the lowest performance compared to other classes was observed in the SCC class, with an AUC of 0.89 for this class. Although this value is acceptable and indicates a high classification success, it can be said that the model struggles more in this class due to factors such as SCC samples having similar visual characteristics to other skin lesions or class imbalance in the dataset. The study has some limitations. The primary limitation is the use of a publicly available dataset. Expanding the dataset with disease data and metadata from different populations, devices, and experts is crucial. Another limitation is the unbalanced nature of the dataset. While this limitation has been partially overcome, creating a more balanced dataset is essential for future research. Cross-dataset validation on several datasets is planned for future research. Creating a centrally located dataset is among our goals for future research.

## 5. Conclusions

This study proposes a hybrid model based on feature engineering that combines CNN, ViT, FT-Transformer, and attention mechanisms for diagnosing and classifying dermatological images. The proposed CNN+ViT+FT-Transformer+Attention model outperformed other approaches in the literature in terms of AUC value, according to experimental results on a six-class dataset. The AUC value obtained in the proposed model is 96.41%. The findings demonstrate that the simultaneous learning of both local and global features improves diagnostic performance when classifying complex and multi-class skin lesions. Additionally, since dermatological images are used in conjunction with clinical data in real-world applications, the proposed method provides a realistic framework. It is believed that image data alone is insufficient for disease diagnosis.

## Figures and Tables

**Table 1:** Class-wise distribution of the PAD-UFES-20 dataset.

Class	Label	Number of İmages
ACK	Actinic keratosis	730
BCC	Basal cell carcinoma	845
SCC	Squamous cell carcinoma	192
SEK	Seborrheic keratosis	235
MEL	Malignant melanoma	52
NEV	Melanocytic nevus	244
Total		2298

**Table 2:** Hyperparameters and training configurations used during the fine-tuning phase.

Parameter	Value/Method
Batch Size	16
Total Epochs	10
Optimizer	Adam
Weight Decay	1 × 10^−4^
Learning Rate Scheduler	Cosine Annealing
Loss Function	Cross-Entropy Loss
Model Selection Criterion	Highest validation accuracy

**Table 3:** Classification performance of pre-trained models for the image dataset.

Model	Accuracy	Macro Recall	Macro Precision	Macro F1 Score	AUC
ConvNeXt V2 Base	0.7652	0.7652	0.7617	0.7622	0.9462
DenseNet 121	0.6543	0.6543	0.6579	0.6545	0.8981
EfficientNet B4	0.613	0.613	0.6401	0.6168	0.8906
Inception v3	0.6022	0.6022	0.6456	0.6084	0.8933
MobileNetV3	0.6652	0.6652	0.682	0.6653	0.8994
Resnet50	0.6609	0.6609	0.6596	0.6524	0.897
ViT B-16	0.7696	0.7696	0.7618	0.7643	0.9367
ViT B-32	0.7348	0.7348	0.7324	0.7331	0.9196
DeiT B-16	0.7283	0.7283	0.724	0.7241	0.9253
Swin Base	0.7543	0.7543	0.7587	0.7559	0.9395

**Table 4:** Classification performance of pre-trained models for Metadata.

Model	Accuracy	Macro Recall	Macro Precision	Macro F1 Score	AUC
FT-Transformer	0.7152	0.7152	0.7169	0.7144	0.9017
ExcelFormer	0.713	0.713	0.6972	0.6972	0.8919
SAINT	0.7087	0.7087	0.6868	0.6861	0.8916
GRANDE	0.7065	0.7065	0.7018	0.6974	0.8686
PTaRL	0.6717	0.6717	0.648	0.6278	0.8843
TabTransformer	0.6239	0.6239	0.5858	0.5903	0.8351

**Table 5:** Performance analysis of the proposed hybrid model on machine learning classifiers.

Model	Accuracy	Macro Recall	Macro Precision	Macro F1 Score	AUC	MCC
Extra Trees	0.8522	0.8522	0.853	0.8486	0.9641	0.7983
Random Forest	0.85	0.85	0.8484	0.8443	0.9603	0.7953
Support Vector Machine (SVM)	0.8435	0.8435	0.8475	0.8361	0.9585	0.7881
Gradient Boosting	0.8326	0.8326	0.8266	0.8249	0.9525	0.7702
Logistic Regression	0.813	0.813	0.8075	0.8087	0.9521	0.7435
XGBoost	0.8435	0.8435	0.8391	0.8376	0.9507	0.7822
Light GBM	0.8543	0.8543	0.8535	0.8499	0.9476	0.8007
Stochastic Gradient Descent (SGD)	0.7891	0.7891	0.7915	0.7892	0.9396	0.7126
Ridge Classifier	0.8	0.8	0.7932	0.7914	0.9304	0.7248
K Nearest Neighbour	0.837	0.837	0.8366	0.833	0.9241	0.7779
Linear Discriminant	0.763	0.763	0.7628	0.762	0.9186	0.675
Naive Bayes	0.7848	0.7848	0.8224	0.7967	0.8945	0.7141
Decision Tree (DT)	0.7804	0.7804	0.7776	0.7761	0.836	0.7006

**Table 6:** Results obtained by adding the CBAM block.

Model	Accuracy	Macro Recall	Macro Precision	Macro F1 Score	AUC	MCC
Extra Trees	0.837	0.837	0.837	0.8303	0.9617	0.7789
Random Forest	0.8304	0.8304	0.8278	0.8214	0.9608	0.7698
Support Vector Machine (SVM)	0.8391	0.8391	0.8431	0.8334	0.9556	0.7833
Gradient Boosting	0.8065	0.8065	0.8092	0.8034	0.946	0.7365
Logistic Regression	0.8304	0.8304	0.831	0.8222	0.9538	0.7686
XGBoost	0.8196	0.8196	0.8186	0.8154	0.9523	0.754
Light GBM	0.8304	0.8304	0.8297	0.826	0.9521	0.7691
Stochastic Gradient Descent (SGD)	0.8174	0.8174	0.8195	0.8051	0.9496	0.7521
Ridge Classifier	0.8087	0.8087	0.8048	0.7997	0.9279	0.7373
K Nearest Neighbour	0.8261	0.8261	0.8318	0.8235	0.924	0.7648
Linear Discriminant	0.7761	0.7761	0.7801	0.7758	0.9264	0.6947
Naive Bayes	0.787	0.787	0.8161	0.7958	0.8982	0.7151
Decision Tree (DT)	0.7652	0.7652	0.7661	0.7639	0.8372	0.6803

**Table 7:** Literature Review.

Papers	Methods	Number of Classes	AUC (%)	Accuracy (%)
Javed et al. [15]	CovnMixer	6	94	-
Azeem et al. [16]	InceptionV3	3	-	96
Bhargavi and Balakrishna [17]	CNN	6	-	96.17
Oztel et al. [18]	ResNet18	6	-	74.62
Wang et al. [19]	DenseNet121	6	98	91.86
Khan et al. [20]	EfficientNetB3-XGBoost	6	83	78
Sathvika et al. [21]	AlexNet	5	-	96.87
Bouzon et al. [22]	MetaBlock	6	-	70.2
Proposed Model	CNN+ViT+Attention+FT-Transformer	6	96.41	85.22

## Data Availability

The original data presented in this study are openly available in Skin Cancer (PAD-UFES-20) data card in Kaggle at https://www.kaggle.com/datasets/mahdavi1202/skin-cancer (accessed on 16 March 2026).
